# Recent Advances on the Prevention and Management of Rheumatic Heart Disease

**DOI:** 10.5334/gh.1402

**Published:** 2025-02-21

**Authors:** Jiawen Zhang, Songhao Jia, Yuhe Chen, Jie Han, Hongjia Zhang, Wenjian Jiang

**Affiliations:** 1 Department of Cardiac Surgery, Beijing Anzhen Hospital, Capital Medical University, Beijing 100029, China; 2 Beijing Lab for Cardiovascular Precision Medicine, Beijing 100069, China; 3 Key Lab of Medical Engineering for Cardiovascular Disease, Ministry of Education, Beijing 100069, China

**Keywords:** Rheumatic Heart Disease, Prevention, Vaccine, Surgery, Mitral Valve Repair

## Abstract

The global burden of rheumatic heart disease (RHD) remains substantial, particularly in low-income countries, despite advancements in prevention and management strategies. This article emphasizes the strategies related to primordial prevention, primary prevention, and secondary prevention, including recent advancements in vaccine development, and discusses current challenges in management and future research directions. For treatment, it evaluates percutaneous mitral commissurotomy, mitral valve repair, and replacement, noting the advantages of individualized approaches based on patient conditions. Addressing RHD’s global burden requires equitable access to surgical treatments, robust healthcare systems, and sustainable strategies for prevention and care.

## 1. Introduction

Rheumatic heart disease (RHD) remains a significant global health challenge and a leading contributor to cardiovascular morbidity and mortality (1). As of 2019, it was estimated that 40.5 million people were living with RHD, resulting in approximately 306,000 deaths annually (2). Rheumatic heart disease exhibits significant global and regional disparities, disproportionately affecting low- and middle-income countries. In 2015, the burden of RHD was evidenced by the loss of 10.67 million disability-adjusted life years (DALYs), with a disproportionate impact on regions such as Central Africa, Oceania, and South Asia, where socioeconomic challenges and limited healthcare access exacerbate its prevalence (3). In contrast, RHD has been nearly eradicated in high-income countries, demonstrating that advanced healthcare systems and effective public health interventions can significantly reduce its incidence (4).

The incidence of RHD is also significantly associated with age. In 2019, the age group with the highest prevalence of RHD was 25–29 years, with a significant increase in prevalence among the elderly population (1). However, relative to the RHD global average, prevalence of RHD still poses a serious public health issue in these regions. Moreover, national-level social, demographic, economic, or environmental changes, including liberal immigration policies, can also impact the incidence of RHD (4,5,6). For example, in Germany, a high-income country, the incidence of RHD has not significantly declined over the past 20 years. On the contrary, mortality rates have exhibited a bimodal increase, particularly among younger and older age groups (4). Given this context, a coordinated global effort is imperative to address the multifaceted challenges posed by RHD. This review aims to examine the latest advancements in preventive strategies, diagnostic tools, treatments, and surgical techniques, while emphasizing the ongoing need for a comprehensive care approach in the global fight against RHD.

## 2. Primordial Prevention and Primary Prevention

### 2.1 Primordial prevention

The social determinants of health (SDH), such as income levels, educational opportunities, housing conditions, and access to healthcare services, play a crucial role at the household, community, and national levels in the incidence of acute rheumatic fever (ARF) and RHD (7). While most high-income countries and some low-income countries have significantly reduced the incidence of RHD, the disease persists in many low-income nations and among specific populations in some high-income countries (8). This persistence is largely attributed to health inequities, poverty, and inadequate SDH in these contexts. By addressing these SDH and broader factors like public health education and overall health status, the risk of exposure to group A streptococcus can be reduced. Thereby reducing the risk of developing ARF and its subsequent progression to RHD.

Historically, community-based prevention programs, such as those implemented in Martinique and Cuba, have emphasized health promotion for both the public and healthcare workers (9,10). These programs combined education with surveillance, leading to significant improvements in overall living standards, increased access to healthcare, and a marked reduction in the incidence of ARF. This approach has significantly improved overall living standards, increased access to healthcare, and reduced the incidence of ARF. As living conditions continue to improve and global poverty declines, the levels of education and surveillance are expected to rise correspondingly, potentially leading to a further reduction in the burden of ARF and RHD.

Several observational studies have indicated a significant association between the degree of social overcrowding and the incidence of Group A Streptococcus (GAS) infections, such as GAS-induced skin infections, and ARF (11,12). Therefore, further research is needed to clarify the relationship between social crowding and disease incidence in resource-limited settings. Meanwhile, there are significant differences in risk among different racial and ethnic groups, which may reflect a combination of intergenerational factors, such as the enduring impacts of systemic racism, colonization, and genetic inheritance, contributing to a familial history of RHD (13). It is imperative to investigate and elucidate the variations in RHD incidence across diverse populations, particularly in relation to SDH, genetic predispositions, and intergenerational influences.

Additionally, repeated exposure to GAS and the cumulative effects of the immune response triggered by such exposures are considered key factors in the pathogenesis of ARF (14). In social crowding, poverty, and limited access to healthcare, the likelihood of such repeated exposures is significantly heightened, underscoring the importance of addressing health inequities. Further research is essential to quantify and develop targeted interventions to better understand the impact of reducing GAS exposure on ARF.

### 2.2 Prevention and control of Streptococcus pharyngitis

The primary strategy for preventing RHD involves the prompt identification and treatment of pharyngitis and impetigo caused by GAS infection, thereby mitigating the risk of ARF in high-risk populations (15). For suspected cases, the administration of antibiotic prophylaxis post-symptom onset can significantly reduce the incidence of ARF by up to 70% (16). However, GAS-induced pharyngitis often manifests with mild symptoms, resulting in many patients not seeking medical attention, which undermines the effectiveness of primary prevention efforts for RHD. Additionally, there is a widespread lack of awareness in many communities regarding the connection between pharyngitis and ARF. Consequently, many ARF and RHD patients may have overlooked prior symptoms of pharyngitis (17). On the other hand, research on GAS impetigo remains limited, and there is still ongoing debate regarding its role as a decisive or contributing factor to the development of RHD (18). However, evidence suggests a definitive connection between GAS impetigo and RHD (19).

The administration of antibiotics to treat GAS pharyngitis has demonstrated efficacy in preventing ARF. Nevertheless, prior to antibiotic use, the atypical manifestations of GAS infection necessitate confirmation through diagnostic testing to mitigate the increased medical costs and patient risks, including the development of antibiotic-resistant bacteria (15). An African experiment developed a model to estimate the health impact, expenses, and monetized health benefits of scaling up RHD primary prevention coverage between 2021 and 2030. This model aimed to calculate the benefit-cost ratio and net benefits. The results indicated that expanding primary prevention coverage could reduce the projected age-standardized incidence of RHD in 2030 by 7.6%, preventing 187,200 new RHD cases from 2021 to 2030 (20). Although bacterial cultures can provide high diagnostic accuracy, their implementation is often impractical in many countries due to the extended time required and the demand for substantial resources. Consequently, regions with a high-burden GAS infection necessitate the availability of rapid and highly specific diagnostic tests for effective disease management. Rapid antigen detection tests (RADTs) represent a prevalent clinical technique for diagnosing GAS infections. This diagnostic approach facilitates prompt identification and accurate treatment, mitigating the risk of progression to ARF or RHD. Rapid antigen detection tests are characterized by their rapidity and ease of use. For patients with high-risk pharyngitis, the implementation of RADTs can help reduce antibiotic use, which in turn contributes to cost savings and enhances cost-effectiveness (21). However, the sensitivity and specificity of RADTs remain relatively constrained, underscoring the necessity for performance improvements to establish them as a principal strategy for primary prevention (22).

### 2.3 Vaccine progress

**Figure 1:**
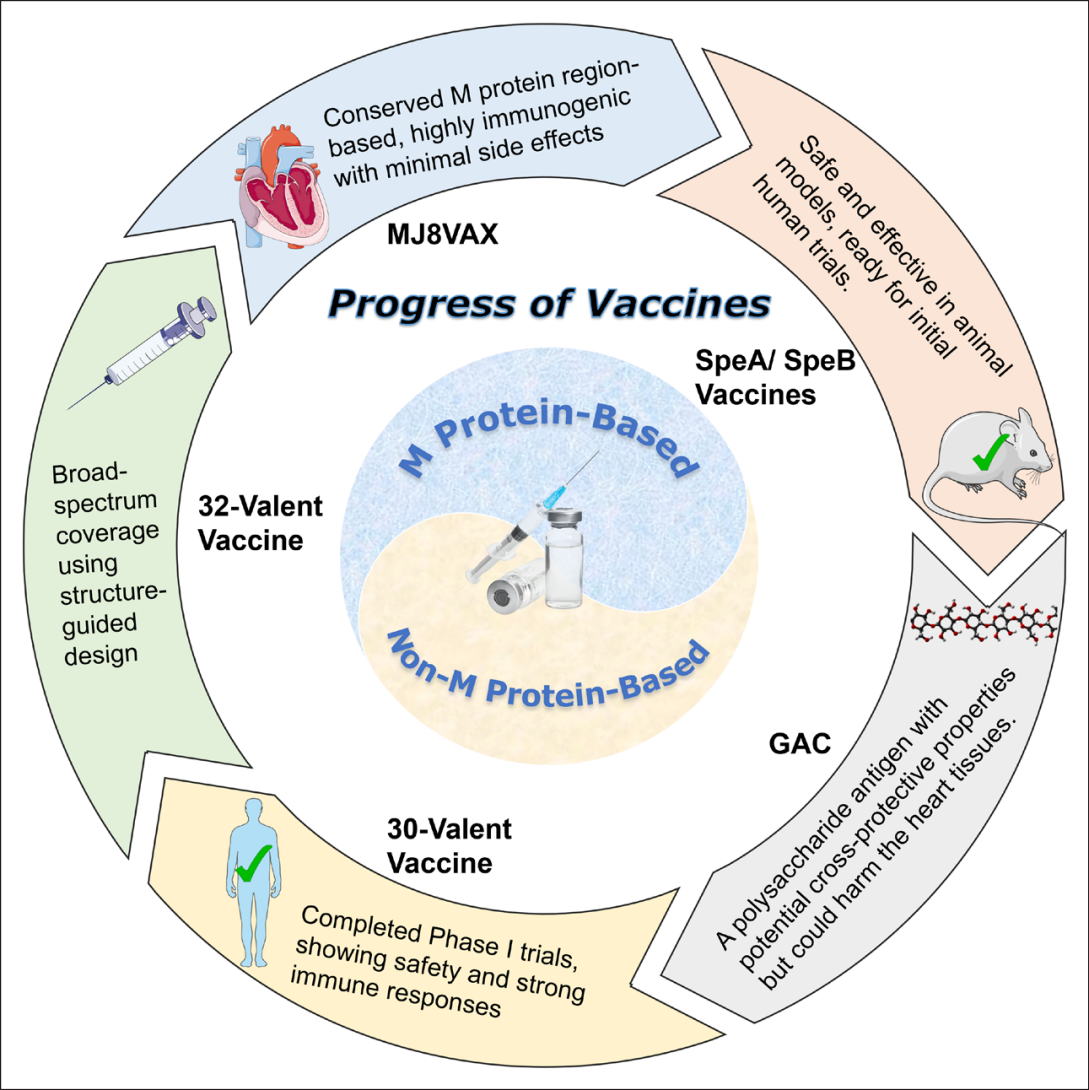
The progress in vaccine research. GAC, Group A Carbohydrate; SpeA, Streptococcal pyrogenic exotoxin A; SpeB, Streptococcal pyrogenic exotoxin B.

Developing a potent and safe GAS vaccine is a promising step in preventing RHD, offering high returns on investment at relatively low costs (23). Developing an effective and safe vaccine for GAS is a promising initiative to prevent RHD, potentially yielding a high return on investment at a relatively low cost. A thorough review of guidelines and literature reveals that research on GAS vaccines dates back to the early 20th century (24). However, despite significant recent advancements, no commercially viable vaccine has yet been developed. The main challenge lies in the potential cross-reactivity between GAS antibodies and human tissues, which could inadvertently trigger RHD (25). The progress in vaccine research is illustrated in Figure 1.

To ensure the safety of GAS vaccines, it is crucial to guarantee that any antigens included in the vaccine do not cross-react with cardiac muscle proteins. In the 1960s, a vaccine trial led to a serious medical incident due to cross-reactivity, where three out of 21 volunteers developed ARF after vaccination (26). The significant increase in ARF cases among vaccinated children forced the cessation of vaccine research. Subsequent research on GAS vaccines has focused on two main antigenic targets: M protein-based and non-M protein-based approaches. The N-terminal region of the M protein can elicit an immune response without cross-reacting with human tissues, a finding first discovered in the 1990s (27).

In studies involving M protein-based vaccines, the M protein is identified as a key virulence factor and the primary target for antibodies against GAS. Encoded by the *emm* gene, this protein is located on the bacterial cell wall surface and can also be used to differentiate clinical GAS strains (28). In theory, the effectiveness of M protein-based vaccines may depend on the match between the selected M proteins in the vaccine and the *emm* types of locally circulating strains (29). Due to the recurrent genetic recombination observed in GAS, a significant genetic heterogeneity occurs among GAS isolates, leading to variations in their protein sequences. Consequently, even to this day, no single protein has been identified to be universally conserved across all GAS isolates (30).

Currently, over 200 distinct types of M proteins exist, each with a unique N-terminal sequence that plays a crucial role in activating immune responses (32). To date, M protein-based vaccine candidates predominantly encompass the purified M protein, multivalent HVR (4,6,8-valent), 30-valent HVR, StreptAnova, and StreptInCor, among others (32). The recombinant 30-valent M protein-based GAS vaccine has already successfully completed Phase I trials, exhibiting robust immunogenicity and tolerability in volunteers without any incidence of autoimmune complications. Despite covering fewer serotypes than the 26-valent vaccine, it still demonstrates considerable promise (33). Another vaccine that underwent clinical trials, MJ8VAX (J8-DT), is a novel acetylated peptide-protein conjugate vaccine comprised of an acetylated peptide antigen (J8) synthesized from the conserved carboxyl-terminal region of the M protein. The results of the clinical trial demonstrated that although the vaccine caused a few mild adverse reactions, it generally exhibited robust immunogenicity and did not induce autoimmune responses (34). J8 peptide epitope’s minimal immunogenicity necessitates its administration alongside adjuvants or vaccine delivery systems. Conjugating J8 with cholesterol boosts antibody production and bactericidal activity, solving the adjuvant issue.

Furthermore, there are significant global epidemiological differences, with GAS emm type diversity being considerably higher in developing countries compared to developed countries (31). As a result, a single vaccine is unlikely to provide comprehensive coverage against all strains in developing countries. Recent research has successfully developed a 32-valent vaccine through structure-guided design, encompassing a more extensive range of M protein types. This progress offers a novel strategy for preventing and treating GAS infections (35). The extensive diversity of M proteins highlights the necessity of researching and developing customized broad-spectrum vaccines based on the epidemiological characteristics of different regions to achieve optimal protective efficacy (31,36).

Research on non-M protein vaccine candidates primarily focuses on carbohydrate and multi-component vaccines. Recent studies have indicated that group A carbohydrate (GAC), a polysaccharide component of the GAS cell wall, can serve as an antigen for cross-protective GAS vaccines. Conjugated GAC antigens can enhance immunogenicity to aid in further vaccine development (37). However, GAC may induce cross-reactivity with heart tissues, necessitating further research (38). In addition to GAS, non-M protein vaccines made from Streptococcal pyrogenic exotoxin A (SpeA) and exotoxin B (SpeB) can elicit a strong immune response without inducing autoantibodies. The safety of this vaccine has been validated in rabbit models, and it is now ready for initial human clinical trials (39).

A hybrid vaccine has been developed combining M protein (p17) and non-M protein (SpyCEP epitope S2) peptides. These antigenic peptides are presented on the surface of Escherichia coli biopolymer particles, thereby stimulating robust antigen-specific antibody responses and cytokine production. This novel approach has been proven feasible in mice model, providing a new strategy for vaccine development (40). However, it is essential to note that vaccines exhibiting immunogenicity do not invariably translate directly into clinical application.

Nevertheless, no non-M protein vaccine has reached the clinical stage (41). All candidate vaccines under consideration directly target the GAS M protein, with the primary antigens being either the highly variable N-terminal fragments or conserved epitopes from the C-terminal region of the M protein (41). Preclinical assessment is hindered by the lack of suitable models, as pharyngitis, the most common symptom, cannot be replicated in rats. The Lewis rat is the most frequently utilized model for evaluating vaccine safety. Other models are used to elucidate GAS infection mechanisms, but a comprehensive model for GAS pathogenesis is still lacking (42). To establish a preclinical platform to assess vaccine safety and efficacy prior to clinical trials, there is an immediate need to develop more precise animal models capable of replicating the spectrum of human streptococcal diseases.

## 3. Secondary Prevention

### 3.1 Early screening

A crucial secondary preventative measure is the implementation of early screening within high-risk populations. Early screening plays a significant role in reducing the subsequent burden associated with RHD. Given that clinically detectable RHD often emerges following the prolonged subclinical phases, early detection through echocardiography and the prompt initiation of secondary prevention can potentially mitigate the overall impact of RHD (43). Consequently, a strong rationale exists for implementing screening initiatives, which necessitate outreach to vulnerable demographics at the grassroots level of communities. This proactive approach aims to detect the early stages of the disease promptly and facilitate interventions to avert subsequent complications.

In recent years, there has been a growing emphasis on handheld echocardiography and proactively screening cases in resource-limited settings. However, high-RHD regions often lack the expertise and infrastructure for universal screening, making non-professional involvement crucial. Accordingly, the 2023 guidelines introduced echocardiographic criteria for non-specialists to identify suspected RHD cases (44). Further evidence from large-scale rheumatic heart disease screening suggests that it may be feasible for non-specialist practitioners to use handheld cardiac ultrasound to perform a single parasternal long-axis view with a sweep of the heart for offsite evaluation (45). This approach enables the detection of RHD across its entire spectrum, from borderline to severe cases, ensuring reasonable detection rates and specificity. Brief training for non-specialist practitioners, supplemented by remote review and expert support, can efficiently scale up RHD echocardiographic screening in high-risk Settings (46). For example, a medical convoy was organized by the Khartoum Medical Students Association, in which five medical students and two recently graduated doctors were trained in the Handheld Echocardiography (HHE) machine for two weeks under the supervision of a senior pediatric cardiologist. They achieved 93% quality in recorded studies, showcasing the feasibility of similar training for nurses and medical assistants (47). Non-experts can leverage record-keeping and real-time data transmission for expert review, allowing seamless remote evaluation of image quality, diagnosis, and candidate selection.

With the advancement of artificial intelligence (AI) technology, AI applications have extended to the screening of rheumatic heart disease. For example, a trial in Uganda demonstrated the potential of AI models in guiding RHD screening. With minimal training, thirty-six novices scanned 50 patients under AI guidance, achieving diagnostic interpretation in over 90% of cases for detecting RHD presence, abnormal mitral valve morphology, and mitral regurgitation (48). Recent studies have shown that machine learning models can be used to identify and quantify mitral regurgitation in pediatric patients through transthoracic echocardiography (49). Additionally, deep learning techniques have demonstrated substantial specificity and accuracy in identifying screening echocardiograms (50). Therefore, applying artificial intelligence to the automatic detection and diagnosis of RHD through ultrasound technology is feasible. It can help reduce the workload of medical professionals and facilitate large-scale screening.

However, implementing large-scale RHD screening among non-experts, while appearing advantageous, also presents certain challenges. It is crucial for non-expert screening to accurately identify the disease to avoid overdiagnosis or false positives, both of which can lead to negative psychological impacts and unsuitable secondary prevention strategies (51). Although short-term training of non-experts is feasible, the lack of standardized protocols and inconsistent outcomes may lead to diagnostic discrepancies.

The early identification of RHD is paramount, facilitated by technological innovations such as handheld echocardiography and AI-driven detection. Despite the considerable potential of these innovations, their widespread implementation presents significant challenges, especially among non-experts. It is imperative to establish standardized training protocols and optimize technological tools to address these hurdles to ensure accurate and accessible screening. These endeavors are crucial in the effective management of RHD and the minimization of associated complications.

### 3.2 Secondary prophylaxis with medication

The current prevalent method of secondary prevention for rheumatic fever cases involving heart disease is the administering of benzathine penicillin G. To validate the theory of antibiotic prophylaxis, a study conducted in Uganda tested a trial named Gwoko Adunu pa Lutino (GOAL) involving children and adolescents aged 5 to 17 with latent rheumatic heart disease. The results showed that the rate of disease progression in the prophylaxis group was 0.8%, significantly lower than the 8.2% observed in the control group, indicating that regular injections of benzathine penicillin G can effectively reduce the risk (52). Another study conducted in Australia found that increased adherence to secondary prophylaxis is associated with a reduced likelihood of ARF recurrence and the effectiveness of RHD prevention (53). Current Australian guidelines also recommend administering intramuscular injections of Benzathine Penicillin G (BPG) every 28 days for five years, following the most recent incidence of rheumatic fever or until the age of 21 (54). The 2023 World Heart Federation guidelines recommend that initiating SAP upon the detection of early RHD may help reduce the overall burden of RHD while also preventing subsequent valve damage (44). From the above, it can be concluded that SAP can effectively reduce the progression and recurrence risks of rheumatic heart disease. And early intervention and the formulation of standardized treatment protocols are essential.

Despite this, the punctual administration of BPG treatment and compliance with SAP continues to be substandard globally, highlighting an ongoing challenge (55,56,57). Based on this, a research team developed an automated SMS service to alleviate the burden on healthcare providers in supporting patients and to improve adherence to secondary antibiotic prophylaxis (58). Indian studies on RHD prevention highlight that secondary prevention is the most cost-effective option for the healthcare system and patients (59). Noteworthily, the argument has long occurred regarding the advantages and disadvantages of intramuscular or oral penicillin. To address the argument, the Intramuscular vs Enteral Penicillin Prophylaxis to Prevent Progression of Rheumatic Heart Disease GOALIE trial is currently underway for the administration of either oral or intramuscular penicillin to evaluate their effectiveness, involving the progression of mild RHD in a pediatric population aged 5–17 over two years (60). This study will validate the efficacy of oral penicillin, potentially influencing current prevention strategies. This is particularly crucial in some low-resource settings, as it can enhance treatment accessibility and patient adherence.

However, some articles posit the necessity for additional research to provide more evidence of the significant benefits of secondary prevention. Currently, the efficacy of most secondary prevention measures is derived from some assumptions drawn from biological plausibility and low-quality data from previous decades (61). Moreover, given that most cases already exhibited RHD at baseline, this constrains the potential advantages of secondary antibiotic prophylaxis (53). Although new evidence offers the efficacy of antibiotics in impeding the progression of lesions, thereby supporting population-based screening. Notably, over half of lesions in an unknown subgroup of children and adolescents spontaneously resolve without any pharmacological intervention (62). Undeniably, secondary antibiotic prophylaxis offers significant benefits for RHD patients. However, further research is needed to validate these studies’ reliability and assess their long-term cost-effectiveness.

## 4. Progress of Treatment

In terms of medication, diuretics, digoxin, beta-blockers, ivabradine, and non-dihydropyridine calcium channel blockers can effectively alleviate symptoms caused by RHD. For patients with moderate to severe mitral stenosis accompanied by atrial fibrillation, vitamin K antagonists (VKAs) are recommended over non-vitamin K antagonist oral anticoagulants (NOACs) (63). According to the latest findings from the INVICTUS study, VKAs are superior to rivaroxaban in reducing the risk of cardiovascular events and cardiovascular death, without significantly increasing the risk of bleeding (64). Therefore, VKAs should be considered the first-choice anticoagulant therapy for patients with rheumatic heart disease-associated atrial fibrillation.

Compared to medication, surgical treatment is more critical and effective. A study based in Tanzania found that interventional therapy showed significantly better outcomes than medication alone for patients with RMS. The mortality rate in the interventional therapy group was notably lower than in the group receiving only medication. Additionally, patients with arrhythmias and multi-valve disease had an increased mortality rate when treated with medication alone (65).

In the surgical treatment of rheumatic heart disease, there are three main approaches: percutaneous mitral commissurotomy (PMC), mitral valve repair (MVP), and mitral valve replacement (MVR). The early stages of the disease are typically marked by mitral regurgitation (MR), in contrast to rheumatic mitral stenosis (RMS) in the later stages. The latest guidelines, namely the 2020 ACC/AHA guidelines and the 2021 ESC/EACTS guidelines, recommend using PMC to treat patients exhibiting symptomatic RMS. Both guidelines stipulate that suitable candidates for PMC should present with severe RMS (valve area ≤1.5 cm²), favorable valve morphology, absence of moderate or severe mitral regurgitation, left atrial thrombus, or severe valvular calcification (66,67). PMC has demonstrated good safety and efficacy over the past 30 years. Even in patients with suboptimal conditions, it can provide significant clinical benefits for most, particularly by delaying the need for surgery and alleviating symptoms (68). A study from South Korea found that the degree of postoperative change in mitral valve area (MVA) can serve as a key indicator of long-term success and risk reduction. Patients with an MVA increase exceeding 0.5 cm² showed a significantly reduced risk of recurrence (69).

When a patient’s native valve condition is insufficient to support PMC, surgical intervention with MVP or MVR is recommended (66,67). But choosing MVP or MVR has been a topic of continued discourse. In terms of the efficacy and safety of MVP and MVR, a meta-analysis of 19 observational studies involving 10,724 patients showed that 3,495 underwent MVP, while 7,229 underwent MVR. The MVP group demonstrated significantly lower early mortality rates and improved long-term survival compared to the MVR group, although it may require more frequent follow-up surgeries (70). Although another study based on data from Taiwan has indicated that MVP significantly reduced the risk of valve-related complications, there are no significant differences in the risk of late mortality and valve-related complications between MVP and MVR. Notably, MVP increases the likelihood of requiring subsequent MV surgeries (71).

Treatment of RHD in pregnant patients requires special attention. Due to pregnancy-induced increases in cardiac output and decreases in vascular resistance, untreated RHD can worsen during pregnancy and lead to a range of adverse maternal and fetal outcomes, including heart failure, pulmonary hypertension, arrhythmias, thromboembolism, and pregnancy-related mortality (72). Overall, MVP is more suitable for pregnant women than MVR due to its advantage of not requiring long-term anticoagulant therapy (72,73). This is because the hypercoagulable state during pregnancy increases the risk of thrombus formation on mechanical valves. If warfarin is used for anticoagulation, it may lead to fetal warfarin embryopathy, and high doses of warfarin are associated with an increased risk of miscarriage and stillbirth (74,75). If low-molecular-weight heparin (LMWH) is used for anticoagulation, it poses several challenges, such as the need for close monitoring of anti-Xa levels, and its efficacy in preventing thrombus formation on mechanical valves may be less effective than warfarin (76,77).

When choosing between bioprosthetic and mechanical valves for MVR, factors such as the patient’s age and regional considerations must be taken into account. Recent advancements in MVR-related research have shed new light on this subject. A retrospective study examined 3,638 patients suffering from rheumatic heart disease and compared the long-term efficacy and safety between bioprosthetic and mechanical valves (78). The results indicated that mechanical valves demonstrated superior long-term outcomes, as evidenced by the reduced rates of all-cause mortality and reoperation, compared to bioprosthetic valves. Mechanical valves are associated with enhanced long-term survival rates, particularly in patients under 65. However, in low-income regions such as Africa, bioprosthetic valves may lead to better outcomes. In these regions, patients with rheumatic heart disease often face poor management of the international normalized ratio (INR) after mechanical valve replacement, leading to severe complications. A common issue is mechanical valve thrombosis, which requires most patients to undergo repeat emergency surgery within less than four years. Due to low adherence to anticoagulation therapy, limited education, financial constraints, and remote geographic locations, the mortality rate associated with these repeat surgeries is exceptionally high (79).

Current guidelines offer a relatively straightforward strategy: performing PMC for patients with favorable valve morphology, with surgical intervention reserved only when PMC is not feasible. However, when it comes to surgical intervention, there are two options—MVP and MVR. The guidelines do not provide detailed recommendations on this choice or consider the complexity of individual patient conditions. For instance, although MVP could reduce early mortality and enhance long-term survival, it may require more frequent follow-up surgeries compared to MVR. Despite the superior long-term outcomes of mechanical valves, the application of mechanical valves still faces considerable challenges in low-income regions due to the inadequate management of anticoagulation therapy and monitoring. The future guidelines should aim to establish more personalized and comprehensive treatment pathways. These should consider the unique attributes of each patient’s valve disease, left ventricular function, and overall health condition, ensuring that the selected intervention yields the most favorable outcome. The goal should be to progress toward a more individualized treatment strategy that considers not only the feasibility of the procedure but also the enduring quality of life and survival rates of patients. A detailed comparison of treatment options is provided in Table 1. Therefore, for patients with rheumatic mitral valve disease, PMC is the preferred treatment when MS has a favorable valve morphology. In instances where PMC is not viable, MVP should be prioritized over MVR whenever feasible due to the lower early mortality rates and better long-term survival of MVP. However, it may require more frequent follow-ups.

**Table 1:** Comparison of treatment options for rheumatic mitral valve disease: PMC, MVP, and MVR.


CATEGORY	PMC	MVP	MVR

**Advantages**	- Minimally invasive. - Faster recovery compared to surgical options. - Suitable for severe mitral stenosis with favorable valve morphology.	- Lower early mortality rates compared to replacement. - Preserves the patient’s native valve and ventricular function. - Enhances long-term survival. - Avoids long-term anticoagulation therapy, especially in pregnancies.	- Standardized surgical procedure with broad indications. Lower rate of reoperation compared to MVP. - Durable solution for severely damaged valves.

**Disadvantages**	- Less effective in extremely severe cases or multivalvular diseases. - Risk of restenosis over time.	- Long-term outcomes remain controversial, with possible need for reinterventions. - Not always feasible for severely damaged valves.	- Requires long-term anticoagulation therapy for mechanical valves. - Bioprosthetic valves have limited durability, especially for younger patients, and may require replacement. - Higher risk of thrombotic complications in low-resource settings with poor INR monitoring.

**Ideal Use Case**	- Patients with severe mitral stenosis but without significant mitral regurgitation or heavy valve calcification.	- Patients with moderate to severe valve disease where repair is feasible, especially young patients or those needing preservation of valve function.	- Patients with severe valve damage unsuitable for repair, or those able to maintain effective anticoagulation management for mechanical valves.


PMC, Percutaneous Mitral Commissurotomy; MVP, Mitral Valve Repair; MVR, Mitral Valve Replacement; INR, International Normalized Ratio.

## 5. Conclusions

This review comprehensively assesses RHD’s worldwide impacts and management strategies, underscoring the critical role of both non-surgical and surgical interventions. Despite progress in primary prevention and vaccine research, RHD continues to inflict significant morbidity and mortality, particularly in settings with limited resources. Surgical interventions, including innovative mitral valve repair techniques, have demonstrated encouraging results in enhancing patient outcomes, but access to these lifesaving procedures remains inequitable. Addressing the global challenge of RHD necessitates a sustained investment in healthcare infrastructure, enhancing diagnostic tools, and developing supportive global health policies. The optimization of intervention strategies and the expansion of access to comprehensive healthcare are crucial measures to ensure equitable health outcomes on a worldwide scale. Future guidelines should aim to establish more personalized and comprehensive treatment strategies.

## Abbreviations

RHD, Rheumatic Heart Disease; GAS, Group A Streptococcus; ARF, Acute Rheumatic Fever; MR, Mitral Regurgitation; PMC, Percutaneous Mitral Commissurotomy; MVR, Mitral Valve Replacement; MVP, Mitral Valve Repair; DALYs, Disability-Adjusted Life Years; RADTs, Rapid Antigen Detection Tests; GAC, Group A Carbohydrate; SpeA, Streptococcal pyrogenic exotoxin A; SpeB, Streptococcal pyrogenic exotoxin B; TR, Tricuspid Regurgitation; INR, International Normalized Ratio; BPG, Benzathine Penicillin G; SAP, Secondary Antibiotic Prophylaxis; HHE, Handheld Echocardiography; AI, Artificial Intelligence; LMWH, Low-molecular-weight heparin; VKAs, vitamin K antagonists; SDH, social determinants of health.
